# Evolving therapeutic pipeline for tumor-infiltrating lymphocytes in metastatic melanoma – a review

**DOI:** 10.3389/fimmu.2026.1801722

**Published:** 2026-03-25

**Authors:** James Fan Wu, Muhammad Abbas Abid, Sharda P. Singh, Virginia Mohlere, Muhammad Bilal Abid

**Affiliations:** 1 Division of Hematology/Oncology, Department of Medicine, Medical College of Wisconsin, Milwaukee, WI, United States; 2 Department of Pathology & Laboratory Medicine, Aga Khan University, Karachi, Pakistan; 3 Texas Tech University Health Science Center, UMC Cancer Center, Lubbock, TX, United States; 4 Department of Hematology/Oncology, The University of Texas Health Science Center at, Houston, TX, United States

**Keywords:** efficacy, metastatic melanoma, safety, tumor microenvironment, tumor-infiltrating lymphocytes

## Abstract

The recent U.S. FDA approval of lifileucel, a non-engineered, autologous tumor-infiltrating lymphocyte (TIL) therapy, for unresectable or metastatic melanoma represents a major milestone for cellular therapies in solid tumors. This review examines the clinical foundation, regulatory development, limitations, and evolution of TIL therapy in metastatic melanoma. Randomized academic data from the phase III M14TIL trial established the efficacy of TIL therapy. The C-144–01 study leading to lifileucel approval demonstrated median duration of response of 36.5 months, median overall survival (OS) of 13.9 months, and estimated 5-year OS rate of 19.7%, a major advance in this anti–PD-1/PD-L1 resistant cohort without effective treatment options. Despite durable responses, classical TIL therapy requires intensive nonmyeloablative lymphodepletion and high-dose interleukin-2 (IL-2), contributing to substantial toxicity and treatment-related mortality that remain barriers to broader implementation. We discuss safety-driven trial terminations related to cytokine augmentation and feasibility or strategic factors underlying discontinuation of programs, underscoring translational challenges beyond biologic efficacy. Engineered TIL platforms aim to improve persistence and reduce systemic cytokine dependence. OBX-115, designed with regulatable membrane-bound IL-15 expression, eliminates the need for IL-2 infusion and has shown early clinical activity. KSQ-001EX uses CRISPR/Cas9 to inactivate SOCS1, while KSQ-004EX additionally targets Regnase-1 to enhance TIL function. Emerging strategies including IL-2–independent expansion platforms, PD-1–edited TILs, and neoantigen-enriched products illustrate ongoing innovation. TIL therapy remains among the most promising strategies in melanoma and solid tumors after immunotherapy failure. Ongoing research aims to optimize cell dose, phenotype, tumor procurement, treatment sequencing, and rational combinations to improve durable benefit.

## Introduction

Recent advances in immune-engaging T-cell therapies have led to significant progress in developing novel treatments across various cancer types, resulting in prolonged patient survival. However, solid tumors are characterized by an immunosuppressive tumor microenvironment composed of regulatory T cells, tumor-associated macrophages, myeloid-derived suppressor cells, inhibitory cytokines, and a dense stromal architecture that collectively impair effective T-cell function. This, coupled with tumor-intrinsic factors such as antigen heterogeneity, on-target off-tumor toxicity, target antigen loss, hypoxia within the tumor bed, and insufficient preexisting tumor-infiltrating lymphocytes (TILs), impede the development of durable immune-engaging T-cell therapies across solid tumors. However, a few bispecific antibodies, TILs, and autologous engineered T-cell receptor therapies have recently been approved in several indications, paving the way for newer horizons in the solid tumor cellular therapy landscape.

**Figure 1:**
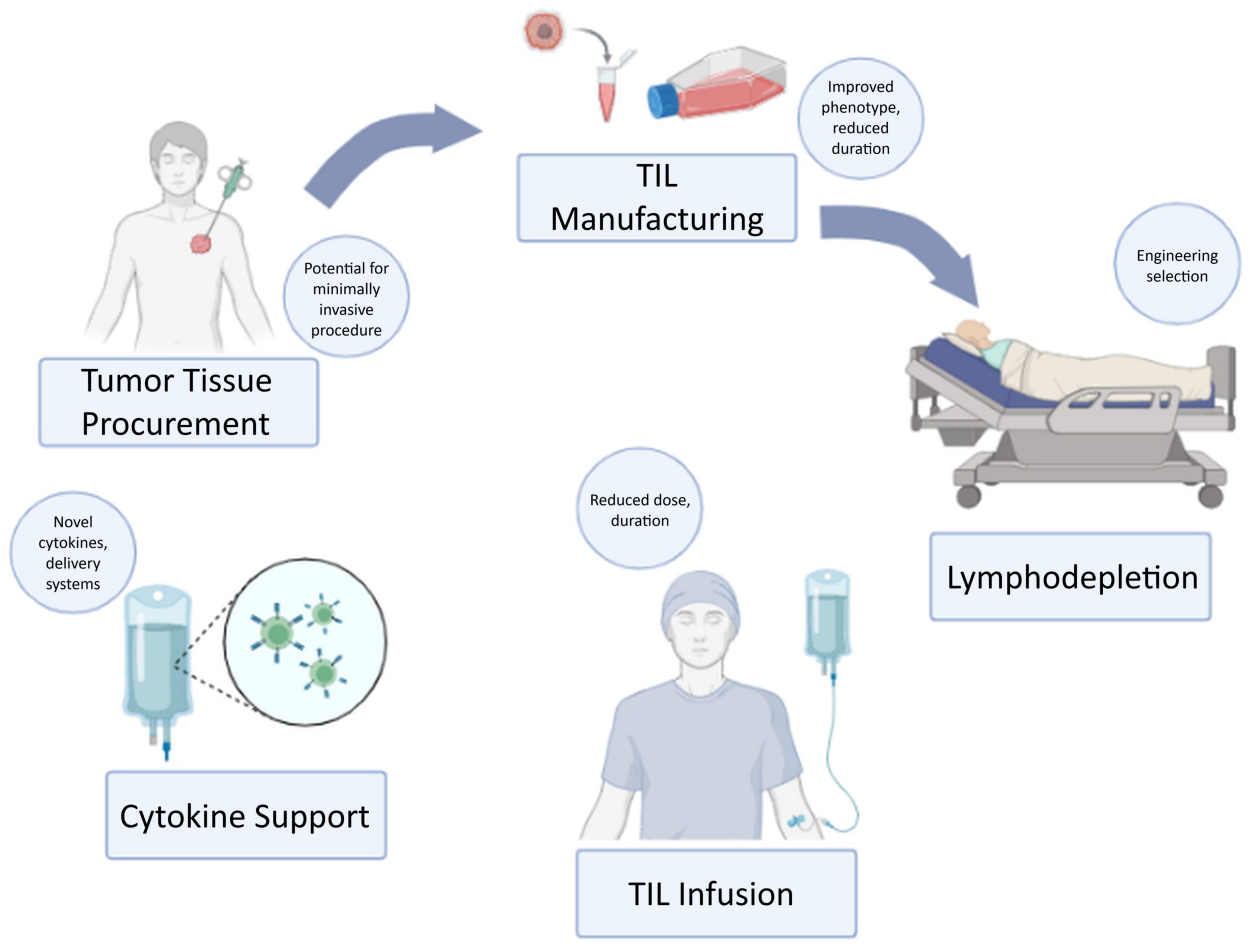
Schematic of the tumor-infiltrating lymphocyte (TIL) therapy process.

Herein, we review the available and early-phase data related to the efficacy and safety of TILs, both approved and in development, in metastatic melanoma (summarized in **Table 1**). TIL therapy traditionally involves the extraction of lymphocytes that directly infiltrate the tumor, followed by TIL manufacturing, high-dose nonmyeloablative lymphodepletion (NMA-LD) chemotherapy, TIL infusion, and high-dose interleukin-2 (IL-2) administration (**Figure 1**). We first examine the clinical foundation and regulatory development of non-engineered TIL therapy, then discuss the limitations of classical platforms that have informed innovation, followed by an overview of promising engineered TIL strategies designed to enhance efficacy and improve the therapeutic index, and conclude with emerging TIL approaches currently in early-phase development.

**Table 1:** Major TIL constructs in therapeutic pipeline for metastatic melanoma.

TIL characteristic	Lifileucel (Iovance)	LM103 (Suzhou BlueHorse)	OBX-115 (Obsidian therapeutics)	KSQ-eTIL (KSQ therapeutics)
Current Status	FDA-approved (accelerated) for metastatic melanoma	Early-phase clinical trials	Early-phase clinical trials	Early-phase clinical trials; limited public data
Development Stage	FDA approved (Accelerated Approval – Feb 2024)	Phase I clinical trial	Phase I/II clinical trial	Phase I/II clinical trial
Indication	Unresectable or metastatic melanoma; prior anti–PD-1/PD-L1 therapy	Advanced/metastatic melanoma and failed standard therapies	Advanced/metastatic melanoma (resistant to anti–PD-1/PD-L1 therapy with or without anti–CTLA-4/LAG-3) and NSCLC	Advanced solid tumors, incl. melanoma (prior anti–PD-1/PD-L1 therapy with or without anti–CTLA-4/LAG-3), NSCLC, HNSCC, CRC, pancreatic, and cervical cancer
Cell Source	Autologous TIL from resected tumor	Autologous TIL from resected tumor	Autologous TIL from resected tumor modified *ex vivo* with synthetic control	Autologous TIL from resected tumor with gene editing
Manufacturing	Autologous TILs cryopreserved and shipped	Details unavailable	Autologous TILs cryopreserved and shipped	Autologous TILs cryopreserved and shipped
Engineering/Technology	Non-engineered autologous TIL expansion	Non-engineered autologous TIL expansion	Pharmacologically regulatable membrane-bound IL-15	CRISPR/Cas9-edited TILs (targeted gene knockout)
Notable Genetic Modifications	None	None	IL-15 regulated by synthetic drug-inducible system	Knockout of genes, *SOCS1* and *Regnase-1*, to enhance TIL efficacy
Mechanism Enhancement	Native TIL expansion + IL-2 post-infusion	Native TIL expansion + IL-2 post-infusion	IL-15 promotes TIL survival & expansion *in vivo*	Enhanced cytotoxicity, persistence, resistance to exhaustion
IL-2 Dependency	Yes – high-dose IL-2 post-infusion	Yes – high-dose IL-2 required post-infusion	IL-2-free; relies on IL-15 expression	No IL-2 in safety lead-in; high-dose IL-2 in subsequent cohorts
Lymphodepletion Regimen	Standard dose cyclophosphamide and fludarabine	Low-dose cyclophosphamide and fludarabine	Low-dose cyclophosphamide and fludarabine (10/11 patients)	Low-dose cyclophosphamide and fludarabine
Latest/updated data (through ASCO 2025)	5-yr Analysis of C-144-01 (n=153): -5-ys OS: 19.7% -mOS: 13.9m -ORR: 31.4% (CR 5.9%, PR 25.5%) -mDOR: 36.5 months	CTR20233999 (n=12): -ORR: 41.7% (5 PR, 2 stable disease, 2 progressive disease) -Disease control rate: 83.3% NCT05971589 (n=4): -ORR: 50% (2 PR, 1 stable disease, 1 progressive disease) -Disease control rate: 75%	Phase-I Single-center (NCT05470283) (n=10): -ORR: 44.4% (2 CR, 2 PR) -Disease control rate: 100% -PFS (24 weeks): 75% Phase-I Multicenter (NCT06060613) (n=11): -ORR: 67% at RP2D (1 CR, 3 PR) -Disease control rate: 100%.	Trial in progress: methods for KSQ-001EX and KSQ-004EX reported at SITC 2024 and ASCO 2025, respectively.
Safety Profile	Cytopenia, capillary leak, infections from lymphodepletion. Common AEs: thrombocytopenia (82.7%), anemia (62.2%), febrile neutropenia (41.7%)	Grade 3–4 TEAEs included leukopenia (100%), lymphopenia/neutropenia (91.7%), fever (83.3%), thrombocytopenia (66.7%), and anemia (33.3%)	No DLTs. Grade 2 CRS (n=2), 5 patients had Grade 3 AEs (pain, ALT/AST increase, hyponatremia, hypokalemia/dehydration).	Not yet reported
Treatment-Related Mortality	7.5% (including deaths due to infections, hemorrhage, organ failure)	None to date	0% so far in single and multi-center studies	Not yet reported
Administration Setting	Inpatient with monitoring for lymphodepletion and cytokine storm	Trial-based; assumed similar	Trial-based; may reduce toxicity with IL-15	Trial-based; assumed similar
Manufacturing Time	~22 days from tumor collection to infusion	Undisclosed	Undisclosed	22 days

TIL, tumor-infiltrating lymphocyte; FDA, U.S. Food and Drug Administration; NSCLC, non-small cell lung cancer; HNSCC, Head and Neck Squamous Cell Carcinoma; CRC, colorectal cancer; ASCO, American Society of Clinical Oncology; OS, overall survival, mOS, median overall survival; ORR, objective response rate; CR, complete response; PR, partial response; mDOR, median duration of response; SITC, Society for Immunotherapy of Cancer; AEs, adverse events; TEAEs, treatment-emergent adverse events; DLTs, dose-limiting toxicities; CRS, cytokine release syndrome.

## Non-engineered TIL therapy

### Academic foundation and randomized evidence

Prior to regulatory approval, academic TIL therapy from Denmark and the Netherlands demonstrated randomized efficacy in the phase III M14TIL trial (NCT02278887), which compared autologous TIL therapy with ipilimumab in patients with advanced melanoma (1). TIL therapy significantly improved median progression-free survival (mPFS) (7.2 months versus 3.1 months; hazard ratio, 0.50; 95% confidence interval, 0.35 to 0.72) and objective response rate (ORR) (49% versus 21%) compared to ipilimumab (1). These findings represented the first randomized evidence supporting TIL therapy in solid tumors. TIL products in this study were manufactured locally at academic centers using traditional pre-rapid expansion and rapid expansion protocols requiring approximately 5–7 weeks of culture with high-dose IL-2. While this trial validated the clinical activity of TIL therapy, the decentralized manufacturing model and prolonged production time posed challenges for scalability and broad implementation.

### Lifileucel (Iovance)

Lifileucel represents the first centrally manufactured, commercially scalable TIL product developed under a standardized 22-day process, enabling regulatory approval and broad clinical implementation. Lifileucel is a non-engineered, autologous TIL therapy, the first of its kind to be approved by the U.S. Food and Drug Administration (FDA) for the treatment of unresectable or metastatic melanoma in 2024 (2). This treatment is approved for patients who have previously been treated with a PD-1 inhibitor or a BRAF inhibitor, with or without a MEK inhibitor (if BRAF V600-positive). The TIL therapy was approved based on the results of the C-144–01 phase-II, open-label, single-arm, multicenter study (NCT02360579) (3, 4). Of the 189 patients initially recruited, 156 were treated with lifileucel. All patients had previously received treatment with an anti–PD-1/PD-1 agent, and 83 (54.2%) had not responded to previous anti–PD-1/PD-L1 therapy. Patients underwent lymphodepletion with cyclophosphamide 60mg/kg once daily for 2 days followed by fludarabine 25mg/m^2^ once daily for 5 days and then received lifileucel infusion. Patients then received high-dose IL-2 infusion every 8–12 hours for up to six doses. The ORR was 31.4%, with a total of 8 complete responses (CRs). The median duration of response (mDOR) was not reached during the initially reported follow-up period. The mPFS was 4.1 months, whereas the median overall survival (mOS) was 13.9 months. While all patients experienced treatment-emergent adverse events (TEAEs), ≥30% had grade ≥3 AEs, including thrombocytopenia (76.9%), anemia (50%), and febrile neutropenia (41.7%). Grade 1/2 infusion-related reactions (IRRs) related to lifileucel were observed in 6 (3.8%) patients. Hypophosphatemia (37%), leukopenia (35%), and lymphopenia (32%) were also reported. Two deaths (arrhythmia and acute respiratory failure) were attributed to NMA-LD, one death (pneumonia) was attributed to NMA-LD and IL-2 infusion, and two deaths (intra-abdominal hemorrhage and bone marrow failure) were attributed to all components of the lifileucel treatment regimen (5). Treatment-related mortality (TRM) was 7.5% (6). The incidence of AEs declined rapidly within 2 weeks after lifileucel infusion.

The updated data, presented at the 2025 American Society of Clinical Oncology (ASCO) meeting with simultaneous publication, reported the 5-year survival outcomes from the C-144–01 study (5, 7). mDOR was 36.5 months, mOS was 13.9 months, and the estimated 5-year OS rate was 19.7%. No new or late-onset AEs related to lifileucel were reported.

### LM103 (Suzhou BlueHorse)

In addition to academic and FDA-approved programs, classical non-engineered TIL platforms continue to be developed across multiple institutions and regions. LM103 is a non-engineered, autologous TIL therapy developed in China that follows the traditional lymphodepletion and high-dose IL-2 backbone, representing independent validation of the conventional TIL approach. Although detailed information regarding the specific manufacturing characteristics of LM103 remains limited, early clinical results have been reported.

The first results in a melanoma cohort from China were presented at ASCO 2025. A Phase-I study (CTR20233999) enrolled patients with metastatic melanoma who had progressed on standard therapies (8). Twelve patients received low-dose NMA-LD (cyclophosphamide 30mg/kg for 2 days and fludarabine 25mg/m^2^ for 5 days), *ex-vivo* expanded LM103 infusion, then high dose IL-2 infusion (200,000 IU/kg for up to 6 doses). The ORR was 41.7% (5 PR, 2 stable disease, 2 progressive disease), and the disease control rate was 83.3%. Grade 3–4 TEAEs included leukopenia (100%), lymphopenia/neutropenia (91.7%), fever (83.3%), thrombocytopenia (66.7%), and anemia (33.3%). The mPFS was not reached, and the most prolonged PFS was 14.14 months. Another group reported the results of treating 4 patients with advanced or unresectable melanoma who failed standard treatment with LM103 (NCT05971589) (9). ORR was 50% (2 PR, 1 stable disease, 1 progressive disease), and the disease control rate was 75%. Bone marrow suppression and fever related to NMA-LD and high-dose IL-2 infusion were reported. Suzhou BlueHorse is also manufacturing several engineered TIL constructs called LM103-Next, Power-TIL LM3030, and TMER-TIL. These assets are in the early phase of development (10), and specific details of these TIL products are not publicly available.

## Limitations of classical TIL platforms and translational constraints

Collectively, academic TIL therapy, centralized commercial lifileucel, and other contemporary non-engineered programs such as LM103 demonstrate reproducible clinical activity across geographic and manufacturing settings. However, their shared reliance on intensive NMA-LD and high-dose IL-2 highlights a common toxicity and logistical framework that has shaped the next phase of innovation.

Although non-engineered TIL therapy can induce durable responses in heavily pretreated melanoma, classical regimens are associated with substantial acute morbidity. NMA-LD followed by high-dose IL-2 contributes to cytopenias, infectious complications, hemodynamic instability, and capillary leak physiology. These toxicities are intrinsic to the platform backbone and have limited broader adoption outside specialized centers.

Efforts to enhance TIL potency through cytokine augmentation have further illustrated the narrow therapeutic window of systemic immune activation. An IL-12–engineered CD8-enriched TIL study in metastatic melanoma (NCT01236573) was terminated due to unexpected toxicities (11). In this trial, TILs were genetically modified using a retroviral vector encoding an inducible NFAT-driven single-chain IL-12 construct intended to enhance tumor-specific activation without exogenous IL-2 support. Despite strong preclinical rationale, clinical translation was limited by systemic inflammatory toxicity attributable to IL-12 expression, as well as a low proportion of durable responses. Similarly, a study evaluating IL-15 administration, which improved anti-tumor effects in murine models, following NMA-LD and TIL infusion (NCT01369888) was terminated because of autoimmune toxicity (12), underscoring the challenges of unregulated cytokine signaling even when designed to improve T-cell persistence.

Importantly, not all TIL program discontinuations were safety driven. An NIH TIL trial (NCT01468818) was closed prematurely due to slow accrual (13), reflecting logistical and operational challenges inherent to individualized cell therapy. Another modified TIL study incorporating engineered feeder cells (NCT01369875) was terminated after failing to meet predefined clinical endpoints of clinical tumor regression and toxicity tolerance (14). In the commercial setting, several programs have been discontinued primarily for strategic or financial reasons. Lyell Immunopharma halted development of LYL845 (NCT05573035), an autologous TIL enhanced with epigenetic reprogramming, after interim data did not meet internal thresholds for continued investment, despite prior regulatory milestones (15–17). Turnstone Biologics discontinued its selected TIL program TBio-4101 (NCT05628883) that recognized patient-specific neoantigens, citing the capital-intensive nature of manufacturing and broader financing constraints (18, 19). Achilles Therapeutics terminated development of ATL001 (NCT03997474) and related neoantigen-enriched TIL platforms after early-phase trials failed to meet commercial viability expectations (20, 21), and Instil Bio discontinued ITIL-168 (NCT05050006, NCT05393635) as part of a strategic portfolio realignment (22–24).

Taken together, these examples demonstrate that discontinuation of TIL programs has reflected a combination of cytokine-related toxicity, manufacturing intensity, feasibility barriers, and evolving corporate priorities rather than intrinsic biologic failure of the TIL platform itself. These constraints have directly informed the development of next generation engineered TIL strategies aimed at improving persistence, modulating cytokine signaling more precisely, and enhancing scalability.

## Engineered TIL therapy

### OBX-115 (Obsidian)

OBX-115 is an engineered, autologous TIL therapy designed with regulatable membrane-bound IL-15 (mbIL15) expression (25). To potentially circumvent the toxicities associated with high-dose IL-2, OBX-115 harnesses cytokine support for TIL expansion and persistence, modulated via a carbonic anhydrase inhibitor, acetazolamide (ACZ), an FDA-approved small-molecule drug (26). The results of the first-in-human, single-center Phase I study (NCT05470283) examining the safety and efficacy of OBX-115 in immune checkpoint inhibitor (ICI)-resistant, advanced melanoma were presented at ASCO 2024 (25). For the 10 patients with unresectable or metastatic melanoma, no dose-limiting toxicities (DLTs) were observed at any dose level. No events of cytokine release syndrome (CRS), immune effector cell-associated neurotoxicity syndrome (ICANS), capillary leak syndrome, treatment-or disease-related mortality, or intensive care unit (ICU) admissions were reported. Grade 3 AEs included increased alanine aminotransferase, abdominal pain, and syncope. In the per-protocol efficacy analysis of 9 patients, ORR was 44.4% (2 complete responses, 2 partial responses). The disease control rate was 100%, and the PFS at 24 weeks was 75%. The results of the multicenter phase I/II Agni-01 trial (NCT06060613) evaluating the safety and efficacy of OBX-115 in ICI-resistant, advanced melanoma was reported at ASCO 2025 (27). Of 11 patients with unresectable or metastatic melanoma, 10 (91%) received low-dose NMA-LD with cyclophosphamide 750mg/m^2^ for 3 days and fludarabine 30 mg/m^2^ for 4 days. There were no DLTs, ICANS, or capillary leak syndrome. Two patients had Grade 2 CRS while 5 patients had Grade 3 AEs (pain, ALT/AST increase, hyponatremia, hypokalemia, and dehydration). No treatment-or disease-related mortality or ICU admission was reported. At the recommended phase-II dose (RP2D) (100 x 10^9^ cell cap of OBX-115 and 500mg/day of ACZ on days 0–6 and 14-20), ORR was 67% [1 CR, 3 partial responses (PR)]. The disease control rate was 100%, and the mDOR was not reached.

Like OBX-115, GT201 which is manufactured by Grit Biotechnology, also employs mbIL15 and is being studied across multiple advanced solid tumors (28). However, preliminary data only reports two melanoma patients without disaggregated melanoma-specific outcomes (29). For the entire cohort, ORR was 55.9% (1 CR, 4 PR, 2 stable disease) and disease control rate was 77.8%. No grade ≥3 adverse events related to GT201 treatment were observed.

### KSQ-eTIL (KSQ)

KSQ-001EX (NCT06237881) and KSQ-004EX (NCT06598371) are engineered, autologous TIL therapies currently being studied in phase I/II trials. KSQ-001EX uses a CRISPR/Cas9 gene editing platform to inactivate the *SOCS1* gene while in KSQ-004EX, both the *SOCS1* and *Regnase-1* genes are inactivated to enhance the TIL efficacy. Both KSQ-001EX and KSQ-004EX demonstrated enhanced anti-tumor functionality *in vitro* and *in vivo* pre-clinical models (30–34). Inactivation of *SOCS1* enhances TIL engraftment, persistence, and the formation of central memory. Similarly, inactivation of *Regnase-1* increases the formation of stem cell memory T-cells and decreases TIL exhaustion. Together, inactivation of *SOCS1* and *Regnase-1* enhance tumor accumulation and tumor-killing functionality of the TIL product. Both gene knockout trials are currently underway in patients with advanced solid tumors including melanoma. For inclusion, patients with melanoma must have progressed on prior systemic therapy for unresectable/metastatic disease after anti–PD-1/PD-L1, with or without anti–CTLA-4/LAG-3 antibodies. Both first-in-human trials are in progress with the first patient dosed with KSQ-001EX in June 2024 (35, 36), and the first patient dosed with KSQ-004EX in April 2025 (37, 38). Patients received low-dose NMA-LD (cyclophosphamide 750mg/m^2^ for 3 days and fludarabine 30mg/m^2^ for 4 days). During the initial dose escalation cohorts, patients are not given IL-2, though subsequent cohorts may incorporate IL-2 dosing.

## Other TILS on the horizon

HS-IT101 is an autologous, unmodified TIL-ACT product developed by Sino-cell Biomed, which is recruiting for its single-arm, multi-center, open-label phase-I clinical trial in advanced solid tumors including melanoma (NCT06342336) (39). HS-IT101 has an expedited manufacturing time of 14 days (40). The Phase I trial will test the safety and preliminary efficacy of low-dose NMA-LD and IL-2 infusion. IOV-4001 (NCT05361174) is another genetically modified TIL developed by Iovance Biotherapeutics. It uses TALEN gene editing technology to disrupt the PD-1 gene in TILs (PDCD-1 gene knockout), potentially enhancing their ability to target and kill tumor cells. Currently, IOV-4001 is an ongoing phase I/II clinical trial in patients with advanced melanoma and non-small cell lung cancer (41), with the first patient dosed in October 2022 (42). Patients will be treated with NMA-LD, IOV-4001 infusion, and a short course of high-dose IL-2. To date, no results have been reported. An academic group from Denmark led by Inge Marie Svane is also investigating PD-1 knockout in TILs but through CRISPR/Cas9 gene editing (NCT06783270) (43). Preliminary data as of November 2025 reported four patients received the CRISPR-edited TIL infusion products, but clinical efficacy data is still pending (44). AGX148, a double-positive CD8 TIL developed by AgonOx using NMA-LD and decremental IL-2 doses, is also being studied as a single agent, as well as in combination with siRNA modulation of PD-1 (AGX148/PH-762), in patients with advanced solid tumors, including those with advanced melanoma (NCT05902520) (45). Additional TIL products currently in early phase pipeline include NEXTGENTIL-ACT (NCT05141474) (46) that recognizes patient-specific neoantigens and NeoTIL-ACT (NCT04643574) (47) that is enriched for tumor antigen specificity, with varying doses of NMA-LD and IL-2. GC101 from Juncell Therapeutics introduces an IL-2–independent TIL strategy combining low-intensity lymphodepletion with hydroxychloroquine and has shown encouraging early clinical signals, supporting ongoing phase II development in melanoma (48–50). Moffitt Cancer Center is investigating a CD40L- and 4-1BBL–augmented TIL platform (NCT06961357) grounded in preclinical mechanistic data demonstrating improved T-cell persistence and metabolic fitness, although clinical efficacy data are not yet available (51, 52). Anavean is testing TIL in combination with ANV419, an engineered IL-2 variant designed to minimize the toxic side effects associated with traditional IL-2, showing promising early phase 1 efficacy signals with a phase 2 trial recruiting (NCT06630611) (53–55).

## Conclusion

TIL therapy represents a major advance for patients with advanced melanoma, particularly in the setting of progression after immune checkpoint inhibition. Durable responses observed with lifileucel and other non-engineered platforms validate the biological potential of autologous tumor-reactive lymphocytes in this disease. However, the current treatment paradigm remains intensive and is associated with substantial toxicity. The requirement for NMA-LD and high-dose IL-2 contributes to significant acute morbidity, including cytopenias, infectious complications, capillary leak physiology, and treatment-related mortality reported in contemporary studies. Although these risks are acceptable in carefully selected patients treated at specialized centers, they remain a major barrier to broader implementation.

From a clinical perspective, improving the therapeutic index of TIL therapy is a central priority. Strategies under investigation include reducing the intensity of lymphodepletion, eliminating or modifying IL-2 support, engineering TILs to enhance persistence without systemic cytokine exposure, and identifying biomarkers to better select patients most likely to benefit. From a basic science standpoint, unresolved challenges include optimizing T-cell phenotype and memory differentiation, preserving stem-like populations during manufacturing, understanding the impact of prior therapies on TIL fitness, and defining mechanisms of resistance and relapse.

Melanoma has served as the prototypical model for TIL development due to its high mutational burden and intrinsic immunogenicity. Lessons learned in melanoma, including toxicity management, manufacturing logistics, cytokine modulation, and gene-editing strategies, provide a framework for adapting TIL therapy to other solid tumors, which may have lower neoantigen loads and more suppressive tumor microenvironments. Thus, melanoma represents both the proving ground and the translational bridge for expanding TIL therapy across solid malignancies.

Continued refinement of conditioning regimens, cytokine support, genetic engineering, and patient selection will be essential to reduce treatment-related risk while preserving durable anti-tumor efficacy. With these advances, TIL therapy has the potential not only to remain a cornerstone of advanced melanoma management but also to inform the next generation of cellular immunotherapies for solid tumors more broadly.
